# Late Cross-Modal Biases in Neural Spatial Representations Revealed by EEG Decoding

**DOI:** 10.1523/JNEUROSCI.0242-26.2026

**Published:** 2026-09-04

**Authors:** Zak Buhmann, Amanda K. Robinson, Jason B. Mattingley, Reuben Rideaux

**Affiliations:** ^1^School of Psychology, The University of Sydney, Camperdown, NSW 2050, Australia; ^2^School of Psychology, The University of Queensland, St Lucia, QLD 4067, Australia; ^3^Queensland Brain Institute, The University of Queensland, St Lucia, QLD 4067, Australia

**Keywords:** audiovisual perception, causal inference, cross-modal interactions, hierarchical processing, multisensory integration, spatial ventriloquism

## Abstract

During audiovisual perception, spatial information from vision and audition is combined, often producing biases such as the ventriloquist effect. While these interactions are well documented behaviorally, it remains unclear when cross-modal information begins to alter modality-specific spatial representations in the brain. Here we used cross-generalized inverted encoding modeling of electroencephalography (EEG) data to track the temporal evolution of spatial representations during a spatial ventriloquist task. Human participants (both sexes) localized audiovisual stimuli with horizontally offset auditory and visual components. Decoders trained on unisensory EEG responses were applied to audiovisual trials to estimate whether and when spatial representations of one modality were biased toward the other. Behaviorally, participants integrated cues but showed slight visual overweighting. Neural decoding revealed robust spatial representations for both modalities, with early unisensory encoding remaining unaffected by cross-modal input. Cross-modal biases emerged only later (from ∼200 ms onward), within a generalized representational window, and occurred sequentially: auditory representations were biased earlier than visual. These findings indicate that multisensory spatial integration arises via recurrent feedback during later processing stages, while early sensory-specific representations remain independent, providing a neural basis for the temporal dynamics underlying the ventriloquist effect.

## Significance Statement

Understanding how the brain integrates spatial information across the senses is central to theories of perception, yet the timing of cross-modal influences on modality-specific neural representations remains unclear. Using electroencephalography and cross-generalized inverted encoding models, we tracked auditory and visual spatial representations with millisecond precision. Early spatial representations remained unisensory despite synchronous audiovisual stimulation. A shared, generalized spatial representation emerged at ∼200 ms, after which cross-modal biases appeared first in auditory and later in visual codes. These results show that spatial integration is implemented through late, feedback-driven processes operating on initially independent sensory estimates, resolving a long-standing discrepancy between early multisensory interactions and late spatial ventriloquism.

## Introduction

Perception depends on transforming physical signals from the world into neural representations. Because sensory signals often share a common external source, the brain integrates information across modalities, resolving ambiguities and enhancing perceptual precision. A canonical example is the spatial ventriloquist effect, in which perceived sound location is biased toward visual input (Bertelson and Radeau, 1981).

A central challenge in multisensory perception is determining whether auditory and visual cues arise from a common source and should be integrated or from separate sources and processed independently (Rideaux and Welchman, 2018; Rideaux et al., 2021). The brain is thought to resolve this via Bayesian causal inference, using spatial location to judge whether cues share a cause (Rohe and Noppeney, 2015); this decision depends on spatial and temporal correspondence and prior expectations (Van Wanrooij et al., 2010). When disparities are small, signals undergo forced fusion and are integrated automatically, each modality weighted by its relative reliability (Alais and Burr, 2004). This raises the question of where and when spatial biases emerge in neural representations.

Neuroimaging has begun to identify cortical loci of audiovisual causal inference. Mihalik and Noppeney (2020) applied multivariate decoding to fMRI data while participants judged whether synchronous auditory and visual signals at matched or mismatched locations shared a source. These decisions were primarily decodable from the dorsolateral prefrontal cortex at the top of the hierarchy, where signals are weighted by bottom–up reliabilities and top–down task relevance. Physical spatial congruency, meanwhile, was decoded from frontoparietal cortices, primarily the frontal eye field and intraparietal sulcus, where integrated spatial representations emerge from bottom–up signals and top–down influences, downstream of early sensory processing (Aller and Noppeney, 2019; Ferrari and Noppeney, 2021). Together, these findings suggest the intraparietal sulcus acts as a hub for audiovisual spatial integration, mediating exchange between sensory cortices and conveying feedback from higher-order regions (Scheliga et al., 2023). This raises a key question: are spatial biases implemented only at higher association stages following causal inference, or do they directly alter modality-specific representations?

It seems reasonable to expect integration to follow causal inference, preventing fusion of signals from different sources, consistent with traditional hierarchical models, in which low-level areas encode each modality separately before integration at higher association areas (Felleman and Van Essen, 1991; Mesulam, 1998; Calvert, 2001). More recent perspectives, however, suggest audiovisual processing is distributed across cortical levels, with dynamic interactions across timescales rather than at one stage (Scheliga et al., 2023; Senkowski and Engel, 2024). Early auditory and visual spatial information is handled separately by the planum temporale and primary visual cortex (Bonath et al., 2007; Callan et al., 2015; Mihalik and Noppeney, 2020), yet these primary areas still receive multimodal input despite a “preferred sense” (Hsieh et al., 2012; Liang et al., 2013; Vetter et al., 2014). Audiovisual interactions often appear within 100 ms of onset (Molholm et al., 2002; Senkowski et al., 2011).

Such early interactions challenge the intuition that signals stay independent until a causal decision is made (Mihalik and Noppeney, 2020). Yet amplitude modulations in primary sensory cortices need not imply integration of spatial representations and may instead reflect nonspecific temporal facilitation rather than genuine spatial fusion. Synchronous processing of a nonpreferred stimulus (e.g., auditory input in the primary visual cortex) may boost the preferred-stimulus response without shifting its spatial representation (Rohe and Noppeney, 2016; Mihalik and Noppeney, 2020). Thus, it remains unclear when cross-modal information begins to bias modality-specific representations.

We tested the hypothesis that cross-modal spatial biases emerge only after early unisensory encoding, implemented later by feedback from higher-order areas. Using cross-generalized inverted encoding modeling of electroencephalography (EEG) responses during a modified spatial ventriloquist paradigm, we tracked when audiovisual stimuli alter modality-specific spatial codes, training decoders on unisensory data and testing them on audiovisual responses. Early auditory and visual spatial representations remained unisensory, with perceptual biases emerging later via sequential cross-modal influences—supporting models in which spatial integration arises through recurrent feedback rather than early sensory fusion.

## Materials and Methods

### Participants

Forty-eight human adults were recruited in return for payment (*M* = 24.48 years; min, 18 years; max, 54 years; 36 females; 46 right-handed). The study was approved by The University of Queensland Human Research Ethics Committee, and informed consent was obtained from all participants. Participants self-reported no neurological or psychiatric disorders and had normal visual acuity (assessed using a standard Snellen eye chart).

### Materials and procedure

The experiment consisted of three conditions, in which the presented stimuli were either visual, auditory, or combined (audiovisual). The order in which conditions were presented was counterbalanced across participants. Before the main task, participants completed two practice rounds each of the auditory and visual conditions. During the practice, participants were directly shown the correct location after each behavioral response. This practice was omitted for the audiovisual condition, because providing specific location feedback might have primed participants to attend to one modality over the other. For the audiovisual task, participants were simply instructed to judge the location of each “stimulus.” The modality was intentionally left ambiguous to avoid a systematic difference in attentional focus between auditory and visual stimuli for the audiovisual condition. The experiment was conducted in a dark, acoustically and electromagnetically shielded room. Stimuli were presented on a 24-inch ViewPixx monitor (VPixx Technologies) with 1,920 × 1,080 pixel resolution and a refresh rate of 120 Hz. Viewing distance was maintained at 54 cm using a chinrest. Stimuli were generated in MATLAB v2021b (MathWorks, 2021) using the Psychophysics Toolbox (Brainard, 1997). Auditory stimuli were played through two loudspeakers (Q Acoustics, 3010i) placed either side of the display (25–75 W, 6 Ω). An EyeLink 1000 infrared eye tracker recorded gaze direction at a sampling rate of 1,000 Hz (SR Research, 2009).

### Stimuli

Visual stimuli were Gaussian blobs (0.2 contrast, 16° diameter) presented for 16 ms on a mid-gray background. Auditory stimuli were 100 ms clicks with a flat 850 Hz tone embedded within a decay envelope (sample rate, 44, 100 Hz; volume, 60 dBA SPL, as measured at the ears). Audiovisual stimuli were temporally synchronous combinations of the auditory and visual stimuli but were spatially offset from each other by ±6° with no other changes to stimulus properties. As audiovisual cues are integrated according to their relative reliability (Alais and Burr, 2004), the visual stimuli were intentionally diffuse to equate the reliability of visual and auditory localization. In doing so, we were able to investigate how visual cues bias auditory representations and vice versa. To manipulate spatial location, stimuli were presented from 31 locations along the horizontal meridian of the display. Stimuli were centered on linearly spaced locations from 15° of visual angle to the left and right of the display center. Auditory stimuli were played through two speakers placed equidistantly either side of the display (58 cm apart). The perceived source location of auditory stimuli was manipulated via changes to interaural intensity and timing (Whitworth and Jeffress, 1961; Wightman and Kistler, 1992). Specifically, auditory stimuli toward the edges of the display had marginally higher volume and were played slightly earlier from the nearest speaker, whereas those toward the center had more uniform volume with less delay between speakers, as in our previous work (Buhmann et al., 2026).

### Experimental design

Participants viewed and/or listened to a random sequence of 1 to 20 stimuli from horizontal locations along the display. Stimulus presentations were separated by a jittered interstimulus interval of 500–700 ms. At the end of each sequence, participants were tasked with localizing the horizontal position of the last presented stimulus by clicking on its estimated location with the mouse cursor. Having participants respond only to the last stimulus provided an increased number of stimulus presentations available for EEG analyses while still recording behavioral responses within the limited time frame of the experiment. To minimize eye movements, participants were asked to fixate on a black dot presented 8° above the display center (see Fig. 1 for an example trial). Stimulus modality was either auditory, visual, or audiovisual, and trials were blocked with short (∼2 min) breaks between conditions. Each condition consisted of 12 blocks of one modality, and each block consisted of 10 trials with a pseudorandomized number of 1–20 stimulus presentations, such that each condition yielded a total of 1,250 stimulus presentations. Each block was followed by a feedback display indicating the average accuracy of participants' responses. In the audiovisual condition, accuracy was calculated relative to the visual cue.

**Figure 1.**
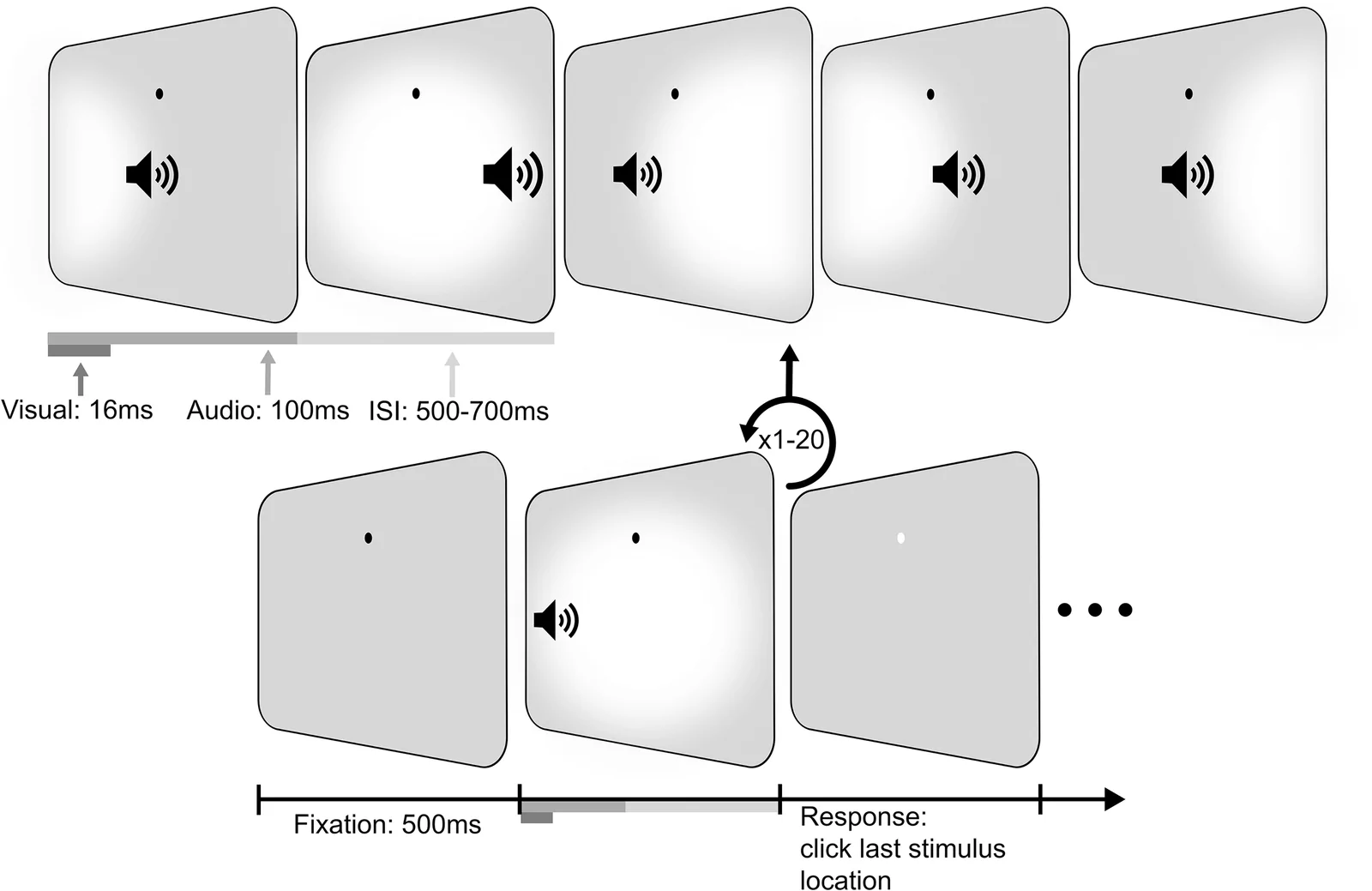
An example trial in the audiovisual condition. On each trial, participants were presented with a random stream of 1–20 auditory and visual stimuli that were spatially localized at one of 31 possible locations (±15°) along the horizontal meridian of the display, while they fixated a dot 8° above the center of the display. The visual and auditory components were always offset by ±6°. The top row displays some possible locations of stimuli. The participants' task was to localize the last presented stimulus as accurately as possible using the mouse cursor. **Note:** The example visual stimuli above have been made more obvious to better communicate the experimental procedure. In the actual experiment, the Gaussian profile was shallower, and each stimulus was more diffuse and harder to localize.

### EEG data preprocessing

EEG data were recorded using a 64-channel BioSemi system at a sampling rate of 1,024 Hz, which was downsampled to 512 Hz during preprocessing. Signals were recorded with reference to the common mode sense, with bipolar electrodes placed above and below the left eye, at the temples, and at each mastoid to monitor for eye movements and muscle artifacts. EEG preprocessing was undertaken in MATLAB using custom scripts and the EEGLAB toolbox (Delorme and Makeig, 2004). Data were high- and low-pass filtered between 0.1 and 45 Hz to remove baseline drifts and high-frequency noise and were baseline-corrected from the average amplitude over the first 100 ms before stimulus onset. Data were then re-referenced according to the average of all 64 channels. Analyses were stimulus-locked, with ERP responses segmented into 1,100 ms epochs from 100 ms before stimulus presentation to 1,000 ms after. No other preprocessing steps or artifact rejection were applied, as these are more likely to reduce statistical power (Delorme, 2023).

### Neural decoding

To characterize the neural representations of stimuli, we used an inverted modeling approach to reconstruct the spatial location of auditory and visual cues based on EEG recordings (Brouwer and Heeger, 2011; Harrison et al., 2023). Analyses were performed separately for visual, auditory, and audiovisual stimuli. We first created a forward model that characterized the patterns of activity in the EEG electrodes given the 31 locations of the presented stimuli. The forward model was used to obtain the inverted model that described the transformation from electrode activity to stimulus location. For the unisensory conditions, we used a 10-fold cross-validation approach where 90% of the data were used to obtain the inverted model on which the remaining 10% of the data were decoded. Cross-validation was repeated 10 times such that all the data were decoded. For the audiovisual condition, we trained the inverted model on the location of stimuli from the unisensory (auditory or visual) data before using it to decode the location of audiovisual stimuli. In doing so, we investigated whether the decoded audiovisual locations were biased by cross-modal influence (e.g., whether a visual-only trained decoder would localize audiovisual stimuli toward the offset auditory stimulus). By training the decoder on auditory or visual stimuli alone, any bias in the decoded audiovisual location should indicate a change in the unisensory representation of stimulus location. To investigate the extent to which the neural representation of location from auditory and visual cues is shared, we performed a cross-generalization decoding analysis between both unisensory conditions (i.e., trained the decoder on unisensory auditory and tested on unisensory visual and vice versa). This allowed us to identify when the representation of spatial location transitioned from a sensory-specific pattern into a generalized form, common to both visual and auditory processing streams.

For the purposes of these analyses, we assume that EEG electrode noise is isotropic across stimulus locations and additive with the signal and as such should not systematically interact with the coding of stimulus locations. Electrodes used in the analysis were obtained from the analysis approach of Buhmann et al. (2026), which used similar stimuli and inverted decoding. Electrodes were selected to maximize the signal-to-noise ratio of location information, which was primarily represented in posterior electrodes for visual and audiovisual stimuli and in central electrodes for auditory stimuli (Fig. 3*A*, inset). The encoding model contained five hypothetical channels, with evenly distributed idealized location preferences between −15 and +15° viewing angles to the left and right of the display center. Each channel consisted of a half-wave rectified sinusoid raised to the second power. The channels were arranged such that an idealized tuning curve of each location preference could be expressed as a weighted sum of the five channels. The observed activity for each presentation can be described by the following linear model:

B=WC+E,

where *B* indicates the EEG recordings (*m* electrodes × *n* presentations), *W* is a weight matrix (*m* electrodes × 5 channels) that describes the transformation from hypothesized channel activity to EEG activity (i.e., the forward model), *C* denotes the hypothesized channel activities (5 channels × *n* presentations), and *E* indicates the residual errors. To compute the inverted model we estimated the weights that, when applied to the data, would reconstruct the channel activities with the least error. Due to the correlation between neighboring electrodes, we took noise covariance into account when computing the model to optimize it for EEG data (Mostert et al., 2015; Kok et al., 2017; Harrison et al., 2023; Rideaux et al., 2023). Specifically, ***B*** and ***C*** were demeaned such that their average over presentations equaled zero for each sensor and channel, respectively. The inverted model was then estimated using a subset of the data, selected through 10-fold cross-validation. The hypothetical responses of each of the five channels were calculated from the training data in each fold, resulting in the response row vector **c***_train,i_* of length *n*_train_ presentations for each channel *i*. The weights on the sensors ***w****_i_* were then obtained through least-squares estimation for each channel:

$$w_{i}=B_{\mathrm{train}}c_{\mathrm{train},i}^{T}(c_{\mathrm{train},i}c_{\mathrm{train},i}^{T}{)}^{-1},$$

where ***B***_train_ indicates the (*m* sensors × *n*_train_ presentations) training EEG data. Subsequently, the optimal spatial filter ***v****_i_* to recover the activity of the *i*th channel was obtained as follows (Mostert et al., 2015):

$$v_{i}=\frac{{\tilde{\Sigma }}_{i}^{-1}w_{i}}{w_{i}^{T}{\tilde{\Sigma }}_{i}^{-1}w_{i}},$$

where ${\tilde{\Sigma }}_{i}$ is the regularized covariance matrix for channel *i*. Incorporating the noise covariance in the filter estimation leads to the suppression of noise that arises from correlations between sensors. The noise covariance was estimated as follows:

$$\hat{\underset{i}{\Sigma }}=\frac{1}{n_{\mathrm{train}}-1}\varepsilon_{i}\varepsilon_{i}^{T},$$

$$\varepsilon_{i}=B_{\mathrm{train}}-w_{i}c_{\mathrm{train},i},$$

where *n*_train_ is the number of training presentations. For optimal noise suppression, we improved this estimation by means of regularization by shrinkage using the analytically determined optimal shrinkage parameter (Mostert et al., 2015), yielding the regularized covariance matrix ${\tilde{\Sigma }}_{i}$. We then used the inverted model to reconstruct the stimulus location from the EEG recordings. The residual error term *E*, and its associated noise covariance, was estimated separately within the training data of each analysis. For analyses trained on auditory-only or visual-only data, the error term was therefore estimated independently for each model and was not pooled across modalities.

To assess how well the forward model captured location information in the neural signal per modality, decoding accuracy was represented in arbitrary units as the similarity of the decoded location to the presented location. To calculate decoding accuracy, a channel response curve was created for each trial and time point, by calculating the dot product between the channel responses and the forward model. The channel response curve was then normalized to between ±1. The predicted location was given by the peak of the curve, while the decoding accuracy was indicated by the value (between ±1) at the true stimulus location. In the audiovisual condition, accuracy was calculated separately relative to the auditory and visual locations. To assess if the decoder showed a bias toward the offset stimuli in the audiovisual condition, we calculated the average difference between the predicted and actual stimulus locations (separately for auditory and visual cues).

### Statistical analyses

Statistical analyses were performed in MATLAB v2025a. For the behavioral analysis, a one-sample Kolmogorov–Smirnov test for each condition revealed that all conditions violated the assumptions of normality. A nonparametric two-sided Wilcoxon signed-rank test was therefore used to test for significant differences in behavioral precision between each pair of conditions. For the neural data, as stimuli were offset by ±6° of visual angle, those that appeared at the edges of the display (i.e., positions −15 to −10° and +10 to +15°) did not have a matched number of left or right offsets. These positions were removed from EEG decoding analyses for the audiovisual condition, averaging 216 presentations out of 1,250. To reduce the potential influence of decision- and motor-related activity, we also removed the final presentation of each trial, where participants responded to the stimulus location, totaling 120 presentations from each condition. Any participants with average decoding performance below 1.5 standard deviations from the group average over the epoch were excluded, though no participants exceeded this threshold.

For decoding performance, a one-sample *t* test was used to compare decoding accuracy to chance (i.e., zero). Next, the summed value of computed *t* statistics associated with each comparison (separately for positive and negative values) was calculated within contiguous temporal clusters of significant values. We then simulated the null distribution of the maximum summed cluster values using permutations (*n* = 20,000) over the sign of the data, from which we derived the 95th percentile threshold value. Clusters identified in the data with a summed effect-size value less than the threshold were considered spurious and removed. To compare the onset of significant decoding accuracy or bias between auditory and visual conditions, we used a leave-two-out jack-knifing approach (1,128 unique combinations for 48 participants) to create subsampled distributions (Robinson et al., 2025). Significant clusters identified in the subsampled data were compared with the 95th percentile threshold value computed from the respective original sample. This yielded a distribution of onset times for each condition. Paired-sample *t* tests were used to test for significant differences.

### Data availability

The behavioral and EEG data and the scripts used for analysis and figure creation are available at https://doi.org/10.17605/OSF.IO/EGH76

## Results

### Behavioral performance

Participants performed well in localizing stimulus location across all conditions. Figure 2*A* illustrates response distributions for a representative participant across conditions. To test for the presence of a systematic bias in participants' responses in the audiovisual condition, we compared the difference between the location of the visual stimulus and the participant's response (Fig. 2*C,D*). Bias values were sign-flipped for leftward auditory offsets so that positive values consistently indicated bias toward the auditory stimulus. To test for successful integration of stimuli in the audiovisual condition, we calculated the bias predicted by maximum-likelihood estimation using the unisensory auditory and visual results (Alais and Burr, 2004). The average bias trended toward being slightly smaller than the maximum-likelihood estimation prediction (*z* = −1.96, *p* = 0.050), indicating participants localized the audiovisual stimulus slightly closer to the visual component relative to the auditory. We found that precision (standard deviation of localization errors) differed across conditions (Fig. 2*B*); precision for auditory stimuli was significantly poorer than precision for both audiovisual (*z* = 3.76, *p* < 0.001) and visual stimuli alone (*z* = 2.11, *p* = 0.035), while precision for audiovisual stimuli was not significantly different than precision for visual stimuli (*z* = 1.47, *p* = 0.142).

**Figure 2.**
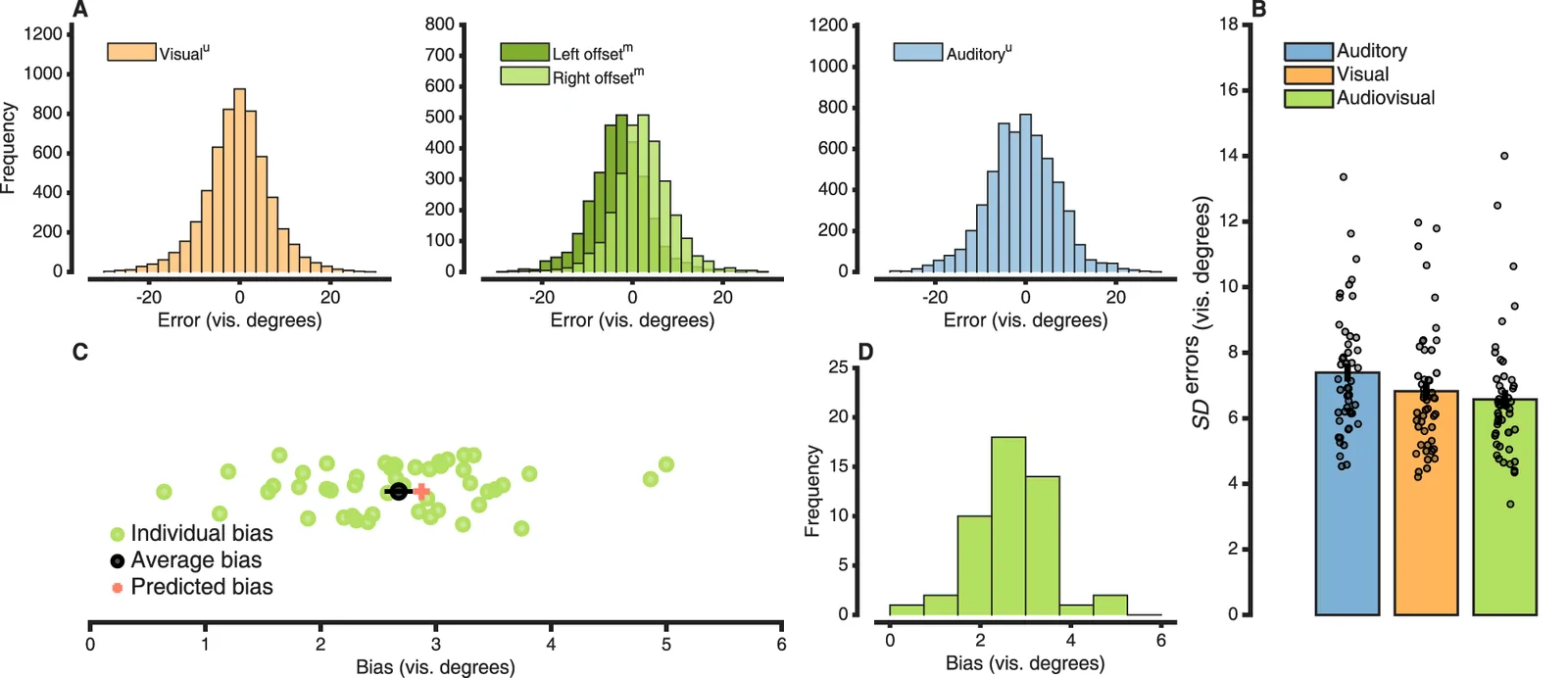
Behavioral performance is significantly influenced by cross-modal oﬀsets. ***A***, Trial-by-trial error between target stimulus location and behavioral response for all conditions aggregated across participants. Error in the multisensory condition is presented relative to the visual stimulus. ***B***, Precision across participants, calculated as the standard deviation of errors for each stimulus condition, relative to the visual stimulus for the audiovisual condition. ***C***, Average bias of behavioral responses across participants in identifying stimulus location for the audiovisual condition. The error bar indicates ±1 SEM. The red cross indicates estimated bias assuming optimal (MLE) integration of unisensory cues. ***D***, Histogram of average bias of behavioral responses across participants for the audiovisual condition.

### Inverted encoding

We used inverted encoding to calculate the spatial decoding accuracy for auditory and visual stimuli in the unisensory conditions (Fig. 3*A*). Decoding emerged at 95 ms for visual stimuli and 145 ms for auditory stimuli and was consistently above chance for both conditions for ∼850 ms. Figure 3*B* shows decoding accuracy for the cross-trained analysis, where the model was trained on unisensory neural activity and tested on audiovisual activity. Spatial location was reliably decoded from 100 ms after stimulus onset for visual stimuli and 170 ms for auditory stimuli, closely matching the results from the unisensory decoding analyses. To test whether the representation of spatial location transitioned from a sensory-specific pattern to a generalized form not unique to either modality, we trained a decoder on the unisensory visual responses and tested it on the unisensory auditory and vice versa. We found reliable cross-generalization from ∼205 to 690 ms following stimulus onset (Fig. 3*C*). 

**Figure 3.**
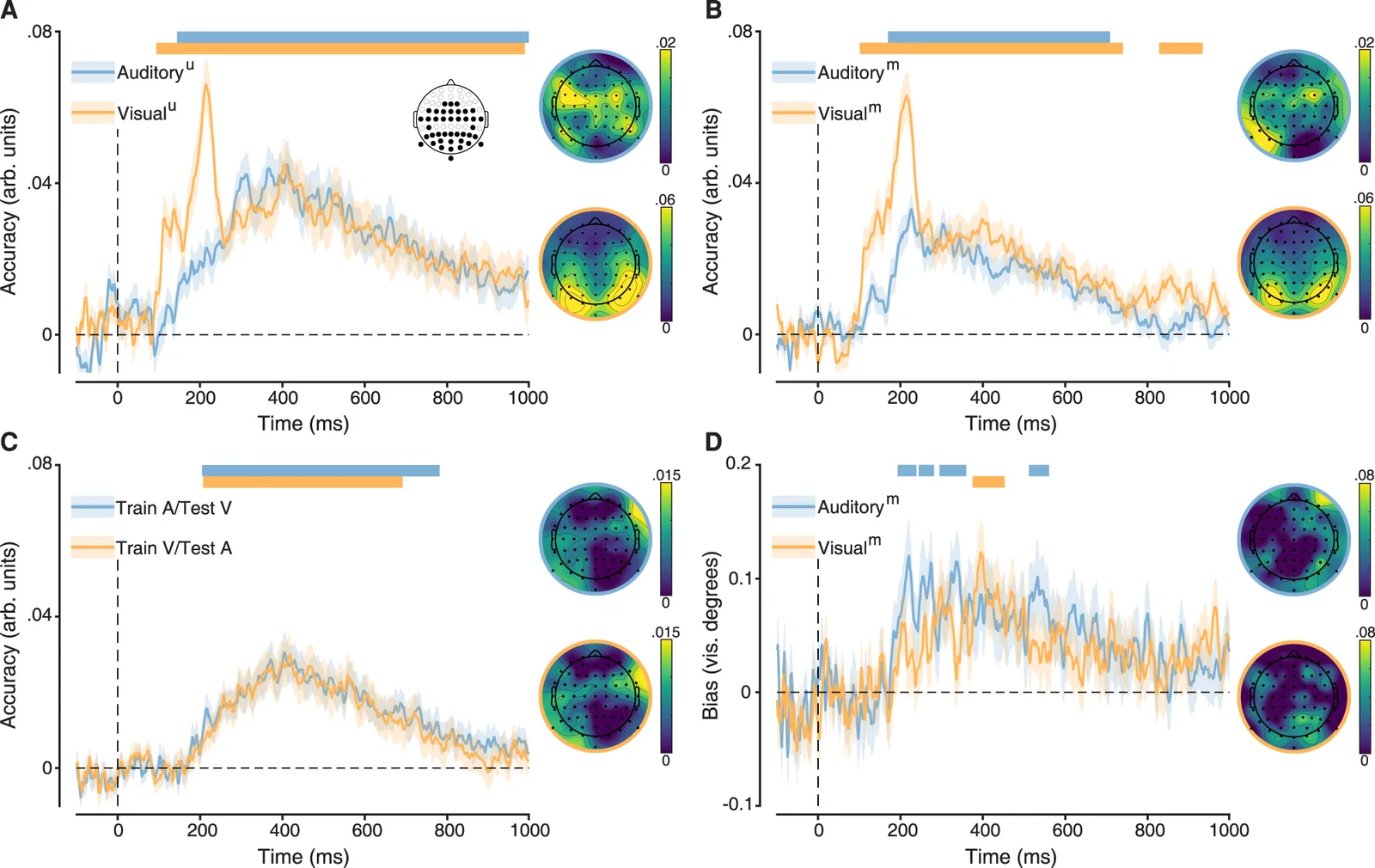
Neural representations of stimulus position are systematically biased by early representations. Accuracy of decoded locations from neural responses to (***A***) unisensory and (***B***) multisensory stimuli. Shaded error bars indicate ±1 SEM. Colored horizontal bars indicate cluster-corrected periods that showed a significant diﬀerence from chance (zero). Right insets show topographic decoding performance across significant clusters. ***C***, Cross-decoding between unisensory auditory and visual conditions to identify modality-independent spatial location information within the neural signal. ***D***, Bias in the decoded location (predicted stimulus location minus actual stimulus location) for audiovisual stimuli. Positive values indicate a greater bias toward the location of the oﬀset stimulus. Right inset shows topographic bias across significant clusters. Inset in ***A*** shows electrode selection for all analyses.

### Neural bias

Having established the temporal profile of spatial decoding, we next tested whether these representations were systematically biased by cross-modal information. To investigate whether the decoder was biased in localizing stimuli toward the offset component, bias was computed as decoder-predicted minus true stimulus location. Biases for left-offset trials were sign-flipped so that positive values reflect bias toward the cross-modal cue (Fig. 3*D*). Auditory-trained decoding showed significant bias beginning at 195 ms and recurring through 560 ms. Visual-trained decoding showed a single significant bias period (∼375–455 ms). Notably, these biases occurred within the period of generalized spatial location identified from the cross-decoding analysis in Figure 3*C*. Paired-sample *t* tests on subsampled data revealed that the onset of cross-generalized decoding emerged significantly earlier than both the auditory bias (*t* = −21.29, *p* < 0.001) and the visual bias (*t* = −100.98, *p* < 0.001). The onset of the auditory bias also arose significantly earlier than the visual bias (*t* = −55.28, *p* < 0.001).

Behavioral bias averaged ∼3°, whereas neural bias estimates were ∼0.25°. The smaller neural bias is likely due to a low signal-to-noise ratio within the recorded neural activity in that the inverted encoding analyses are less sensitive than behavioral measures to condition-specific information. The decoder's trial-to-trial predictions of stimulus location are subject to considerable noise. As the bias is averaged across many trials, this results in an attraction to the mean (zero). Thus, the decoded bias likely underestimates the neural bias.

The neural processes that localize events in space are closely tied to the saccadic eye movements that allow rapid, accurate orienting toward novel stimuli (Sparks, 2002). To examine the potential influence of eye movements toward presented stimuli, we reanalyzed the data using the four electrooculography (EOG) electrodes, which were positioned above and below the left eye and at the temples (Fig. S1). After training on audio and visual trials, the decoder was able to discern auditory and visual stimulus locations significantly above chance. Decoding was also biased for both auditory and visual stimuli at ∼400 ms after stimulus presentation, suggesting that participants' saccades were influenced by both visual and auditory cue location. Critically, however, the early auditory bias observed at 200 ms in the main analyses was not present. To help disentangle neural activity relating to preparatory eye movements from activity associated with stimulus location, we reanalyzed the data using a more stringent preprocessing method with artifact rejection via independent component analysis (SASICA; Chaumon et al., 2015). We found the same pattern of results as in our original analyses (Fig. S2). Given this, and because the main analyses did not use frontal electrodes to minimize ocular muscle artifacts, we believe eye movements are unlikely to underlie the key decoding result.

## Discussion

The current study aimed to examine how spatial representations of stimuli are influenced by cross-modal input throughout the sensory processing stream. We asked when cross-modal information biases modality-specific spatial representations and whether this provides a neural basis for the ventriloquist effect. Participants integrated audiovisual cues, localizing stimuli near the midpoint between auditory and visual components. Critically, multivariate decoding analyses revealed a neural bias, in which the spatial representations of audiovisual components were biased toward the location of the cross-modal component. The bias occurred within a generalized representational window, suggesting hierarchical processing with distinct modality-specific and multimodal spatial representations. The bias also occurred sequentially rather than synchronously, with auditory representations being biased before visual representations. That the bias occurs sequentially despite appearing within a generalized period suggests a complex and hierarchical processing network for spatial information, perhaps with multiple representations of a stimulus.

Participants perceived the source of the event to be roughly midway between the offset auditory and visual stimuli, suggesting successful integration of the multimodal spatial information (Bertelson and Radeau, 1981; Alais and Burr, 2004; Bruns, 2019). However, the perceived location was not consistent with maximum-likelihood estimates and showed a marginal deviation toward the visual component. Response precision for audiovisual stimuli exceeded that for auditory but not visual, which may indicate more frequent attention to the visual modality. It is noteworthy, however, that this pattern of results is consistent with previous work suggesting the primacy of vision in human observers, such that visual information is overweighted relative to information in other modalities, despite optimal integration (Battaglia et al., 2003; Aller et al., 2015; Meijer et al., 2019). As such, the slight visual preference observed in participants' behavioral responses in the current study may reflect greater reliability or salience of the visual stimuli in audiovisual trials.

Decoding results for both the multisensory and unisensory conditions followed a similar overall profile, suggesting that much of the location information within the neural response to unisensory components is carried over into the multisensory response. Visual stimuli were decoded from ∼100 ms poststimulus onset, whereas auditory stimuli were decoded later, at ∼140 ms. The later emergence of auditory decoding likely reflects the need for cortical computation of spatial cues, whereas visual space is encoded retinotopically in early visual cortex. Indeed, audiovisual stimuli are more likely to be judged as synchronous if the sound occurs after the visual cue (Vroomen and Keetels, 2010, 2020). Because auditory spatial cues (i.e., interaural time and level differences) must be computed (sub)cortically (Whitworth and Jeffress, 1961; Wightman and Kistler, 1992), early A1 responses may not contain spatially separable population patterns and therefore cannot be decoded.

The cross-trained unisensory decoding analyses revealed common activity between the auditory-only and visual-only conditions, which became significant at ∼200 ms poststimulus and peaked at ∼400 ms. This reflects a generalized multisensory spatial representation and is consistent with prior work showing that spatial audiovisual information peaks at ∼200 ms (Bednar and Lalor, 2020; Rideaux, 2024; Buhmann et al., 2026). Within the ventriloquist effect, feedback for spatial localization (i.e., physical cue congruence and causal decisions) has been detected after 200 ms poststimulus onset (Bonath et al., 2007; Rohe and Noppeney, 2016). Early visual decoding (∼100 ms) likely reflects retinotopic activity in V1, whereas the later peak (∼200 ms) reflects distributed spatial representations influenced by feedback (Robinson et al., 2021). The auditory spatial information peaked at ∼400 ms poststimulus in the auditory-only condition, while in the audiovisual condition it plateaued after 200 ms poststimulus. The plateau in the audiovisual condition may reflect a transition from modality-specific to generalized spatial coding, which dilutes sensory-specific patterns. Since the visual information had already peaked prior to the generalized representation, it remained relatively unaffected.

Our key analysis investigated whether a neural bias was present within the processing of audiovisual stimuli. Both auditory-trained and visual-trained decoders showed systematic shifts toward the cross-modal cue. As the bias emerged only after the onset of the generalized representation, early modality-specific spatial codes appear to remain unaffected by cross-modal input. Though evidence for audiovisual integration has been found much earlier (i.e., pre-150 ms poststimulus onset; Senkowski et al., 2011; Zhao et al., 2018), these early effects primarily reflect amplitude modulations rather than changes in feature-selective spatial codes (Talsma et al., 2010; Aller et al., 2015; Mihalik and Noppeney, 2020). The integration of stimulus feature information may occur relatively late in perceptual processing, with the localization of an audiovisual stimulus being finalized during a stage in which the spatial information is common to both audition and vision.

A late bias in the neural representation of spatial information is consistent with theories of a hierarchical processing network, where the integration of spatial information occurs within higher-order areas influenced by feedback (Friston, 2005; Mihalik and Noppeney, 2020). Causal inference signals from prefrontal regions may feed back to parietal areas, where spatial estimates are integrated (Kayser and Shams, 2015; Mihalik and Noppeney, 2020). Visual and auditory representations remain separate in early sensory cortices before converging in higher-order regions such as the intraparietal cortex (Callan et al., 2015; Scheliga et al., 2023). Maintaining separate sensory representations before integration preserves accuracy and provides a mechanism to detect inconsistencies that would be lost if unisensory information were discarded prematurely. As the bias in the auditory spatial information also occurred significantly earlier than the bias in the visual representation, the integration of auditory and visual information may be sequential rather than synchronized. One possibility is that the earlier auditory bias reflects integration occurring as soon as reliable spatial estimates become available. The maintenance of auditory information in working memory seems to involve both visual and auditory cortical pathways, while visual information is maintained exclusively within visual areas (Wolff et al., 2020). Though our results concern immediate perceptual processing rather than working memory, they may reflect a similar pattern. The bias in auditory representations occurs first, which may be due to an early integration with the visual signal. The later visual bias, meanwhile, only occurs once spatial information has entered a general format, as visual information may be maintained in a sensory-specific network isolated from auditory processing areas. Future manipulations of audiovisual timing will be needed to test whether aligning peak representational times produces synchronous rather than sequential biases. We also did not measure which modality participants prioritized when localizing audiovisual stimuli, assuming that individual preferences would not systematically bias group-level results. Interindividual variability in the audiovisual condition may nonetheless reflect individual differences in strategy. Future work should therefore examine how attentional focus shapes the perception and integration of audiovisual stimuli (Talsma et al., 2010).

Localization and gaze orientation are tightly coupled processes (Sparks, 2002), and saccadic eye movements are therefore difficult to suppress entirely in paradigms such as ours. Decoding analyses undertaken on data recorded from EOG electrodes indicated that on at least some trials, participants probably made small saccades toward the auditory and visual stimuli, despite instructions to fixate. These saccades appeared to be slightly biased in the direction of the offset stimulus, supporting the finding that participants perceived audiovisual stimuli as being located between the veridical auditory and visual locations. Similar to results from parieto-occipital electrodes, the decoding bias for both auditory and visual conditions was present at 400 ms poststimulus onset and likely reflected similar processes. Here, the biased representations of stimulus location may influence gaze orientation behaviors or vice versa. Critically, however, the early sequential bias found for auditory stimuli was not present when decoding from EOG electrodes. Visual localization is rapid, whereas auditory location must be computed from interaural time and level differences (Martin et al., 2015; Xiong et al., 2022). This earlier bias may reflect a window in which the neural representation of auditory location is already susceptible to visual input, before auditory information has been integrated in turn to influence the visual representation. The bias observed in the EOG decoding may therefore reflect saccades toward the integrated percept alone rather than toward sensory-specific representations. Despite existing evidence for early multisensory interactions (Molholm et al., 2002; Senkowski et al., 2011; Zhao et al., 2018), here we found spatial representations remained unisensory until later stages. Together, these findings indicate that multisensory spatial integration is implemented via recurrent feedback during a later processing stage, consistent with Bayesian causal inference frameworks in which independent sensory estimates are retained before being merged.
